# 1613. Frailty is Associated with Intestinal Barrier Dysfunction in Aging People Living with HIV

**DOI:** 10.1093/ofid/ofad500.1448

**Published:** 2023-11-27

**Authors:** xiaolei xu, Jiangyu Yan, Yanqiu Lu, Mengru Yang, jing ouyang, yaokai Chen

**Affiliations:** Chongqing Public Health Medical Center, chongqing, Chongqing, China; Chongqing Public Health Medical Center, chongqing, Chongqing, China; Chongqing Public Health Medical Center, chongqing, Chongqing, China; Chongqing Public Health Medical Center, chongqing, Chongqing, China; Chongqing Public Health Medical Center, chongqing, Chongqing, China; Chongqing Public Health Medical Center, chongqing, Chongqing, China

## Abstract

**Background:**

Recent evidence indicates that frailty is prevalent in PLWH compared to those without HIV, especially in the elderly population. However, the underlying mechanisms fundamental to this phenomenon reman unclear. Gut damage and increased microbial translocation are hallmarks of HIV infection, and are known to promote and perpetuate the chronic systemic inflammation seen in PLWTH. Meanwhile, it has been observed that chronic inflammation also contributes to the frailty. Herein, we investigate the association between frailty and intestinal barrier dysfunction in PLWH.

**Methods:**

This cross-sectional study was designed to assimilate and analyze clinical data and results of blood samples in PLWH. According to frailty phenotype assessment, participants were stratified into three groups comprising a frailty, a pre-frailty, and a non-frailty group. We subsequently measured the levels of biomarkers of gut damage, microbial translocation, and levels of inflammatory cytokines to assess the relationship between these biomarkers and frailty.

**Results:**

One hundred and fifty one PLWH over 50 years of age were involved in this study, and were stratified into three groups: 73 patients in the non-frailty group, 61 patients in the pre-frailty group, and 17 patients in the frailty group. Their incidence of anxiety, depression, and stress was calculated to be higher in the frailty group than other two groups (p< 0.05). The concentration of gut damage biomarkers in serum, i.e., levels of regenerating islet-derived protein-3α (REG-3α) and intestinal fatty acid-binding protein (I-FABP), showed significant positive correlations with frailty, and was observed to be highest in the frailty group. On the contrary, no significant association was observed between the hematological biomarkers of microbial translocation with frailty. Additionally, IL-6, IP-10, and TNF-α concentrations in plasma were found to positively correlate with frailty in aging PLWH (i.e., these biomarkers had the highest concentrations in the frailty group).

**Conclusion:**

Our study revealed that the inflammation induced by gut damage may contribute to frailty in elder PLWH.

**Disclosures:**

**All Authors**: No reported disclosures

